# Self-Reported Efficacy of Treatments in Cluster Headache: a Systematic Review of Survey Studies

**DOI:** 10.1007/s11916-022-01063-5

**Published:** 2022-06-27

**Authors:** Sakari Santeri Rusanen, Suchetana De, Emmanuelle Andree Danielle Schindler, Ville Aleksi Artto, Markus Storvik

**Affiliations:** 1 School of Pharmacy, University of Eastern Finland, Kuopio, Finland; 2 Department of Neurology, Yale School of Medicine, New Haven, CT USA; 3 Department of Neurology, Helsinki University Hospital, University of Helsinki, Helsinki, Finland

**Keywords:** Cluster, Headache, Survey, Efficacy, Comparison, Review

## Abstract

**Purpose of Review:**

The use and efficacy of various substances in the treatment of CH have been studied in several retrospective surveys. The aim of the study is to systematically review published survey studies to evaluate the reported efficacies of both established and unconventional substances in abortive and prophylactic treatment of both episodic and chronic CH, specifically assessing the consistency of the results.

**Recent Findings:**

No systematic review have been conducted of these studies previously. A systematic literature search with a set of search terms was conducted on PubMed. Retrospective surveys that quantified the self-reported efficacy of two or more CH treatments, published in English during 2000–2020, were included. Several key characteristics and results of the studies were extracted. A total of 994 articles were identified of which 9 were found to be eligible based on the selection criteria. In total, 5419 respondents were included. Oxygen and subcutaneous triptan injections were most reported as effective abortive treatments, while psilocybin and lysergic acid diethylamide were most commonly reported as effective prophylactic treatments. The reported efficacy of most substances was consistent across different studies, and there were marked differences in the reported efficacies of different substances. The reported order of efficacy is generally in agreement with clinical studies. The findings suggest that retrospective surveys can be used to obtain supporting information on the effects of various substances used in the treatment of CH and to form hypotheses about novel treatment methods. The consistently reported efficacy of psilocybin and LSD in prophylactic treatment indicates need for clinical studies.

**Supplementary Information:**

The online version contains supplementary material available at 10.1007/s11916-022-01063-5.

## Background

Cluster headache (CH) is the most common trigeminal autonomic cephalalgia. In the typical form of the disorder, an extremely severe unilateral headache strikes several times a day, often at precise times, accompanied by ipsilateral autonomic symptoms. Daily attacks may last for hours and occur as a series (cluster cycle) that may last for months, typically followed by a symptomless remission period of months or years. This episodic form (ECH) is the more common form of CH. If the symptomatic period continues for over a year with no remission period lasting more than three months, the disorder is classified as chronic (CCH) (ICHD-3). The etiology of CH is not entirely known, but there are some suspected pathophysiological mechanisms, as well as key neuroanatomical regions known to be involved, such as the posterior hypothalamus [1–3]. Possible hereditary risks for the disorder have also been suggested. Global lifetime prevalence is roughly 1/1000. [1]. There are acute and preventive treatments, as well as transitional (or interim) bridge treatments which are used until preventive treatments have taken effect. In the literature, transitional treatments are often grouped together with preventive treatments [1, 2, 4–7].

Subcutaneously or intranasally administered triptans and high-flow oxygen inhaled through a non-rebreather mask are recommended as the first-line options for acute treatment. There is also some clinical evidence for the acute effectiveness of intranasal lidocaine, cocaine and dihydroergotamine sprays, subcutaneous octreotide, and intravenous somatostatin. [1, 4, 6–8]. There is limited clinical evidence for the efficacy of preventive medication, and complete remission is rarely achieved. Recent recommendations are based on a systematic review of available scientific evidence and the expert consensus on the subject [2, 4]. The most recommended preventive treatment is verapamil, followed by lithium. Topiramate and melatonin are usually recommended as tertiary options [1, 2, 4–7]. There have also been some positive clinical studies on the prophylactic use of galcanezumab, capsaicin, warfarin, methysergide, sodium oxybate, clomiphene, amitriptyline, pizotifen, and LSD-analog BOL-148 [2, 4–6, 9, 10].

Oral regimens of corticosteroids have been used in transitional treatment for decades, and in recent years, local corticosteroid injections near the greater occipital nerve have proven effective in clinical studies. Oral frovatriptan and intravenous dihydroergotamine regimens have also shown some transitional effects in clinical studies [1, 4, 6, 7]. Neuromodulatory treatments can be attempted in certain treatment-resistant patient groups. Invasive treatment methods include stimulation of the occipital nerve, sphenopalatine ganglion, or deep brain structures by a surgically implanted stimulator. Transdermal stimulation of the vagus nerve, a non-invasive option, can also be utilized [1, 4, 6, 7, 11, 12].

In addition to treatments prescribed or administered by a clinician, an estimated one-third of CH patients use complementary or alternative treatment methods such as physical therapy maneuvers, relaxation techniques, acupuncture, herbal medicine or homeopathy, or pharmacological substances. Many of the used substances are illicit and have not been clinically studied in the treatment of CH [13–18]. The use of these various unconventional treatments by CH patients speaks to the limitations of the available conventional treatments [19] and points towards an urgent need to find better treatment options.

In prospective clinical studies, the effects of CH treatments are primarily measured with symptom diaries and other patient reports [20–24]. There are no biological markers to indicate disease improvement or remission, and it is not possible to objectively measure the principal symptom of the disorder and pain [1, 25, 26]. Retrospective surveys, despite their methodological shortcomings and biases, can still be utilized to study and compare self-reported efficacy of treatment options over a long period of time in large patient samples. These surveys also reflect the real-world experiences of the patients, at least, to some extent. If consistent patterns are detected across different surveys (e.g., consistently reported high efficacy of a specific treatment), such information can be used to guide empirical research [27, 28].

## Objective

Several retrospective surveys have inquired about the use and self-reported efficacy of various established medications and unconventional substances in different countries. However no published attempts exist to identify the consistent patterns in the use and self-reported treatment efficacy among these surveys. The aim of this study was to systematically review the surveys and identify if any consistent patterns emerge, focusing on the self-reported efficacy of both established and unconventional substances in abortive and prophylactic treatments of CH. The primary questions we wanted to answer were (1) which treatments have been consistently reported as most effective in the surveys, (2) how consistent the results of the surveys are, and (3) whether the survey results are in general agreement with the findings of randomized controlled trials (RCTs). Secondarily, we evaluated which factors in the designs of the individual surveys could have caused biases, thereby affecting the outcomes of the respective surveys.

## Methods

### Selection of the Studies

The study objectives guided the development of the search terms and the inclusion and exclusion criteria. A PubMed search was conducted in May 2020 and again in December 2020 using the developed search terms (Table 1). Only the English language was used in the search terms. The search was applied to “All Fields,” and the listed search terms were combined with the “OR” function. The produced search function can be found in the Appendix. The selection of the studies was done in collaboration with an information specialist.

**Table 1:** Exact search terms used and the criteria for inclusion

Search terms
“cluster headache” survey
“cluster headache” questionnaire
“cluster headache” census
“cluster headache” poll
“cluster headache” inquiry
“cluster headache” interview
“cluster headache” comparison
“cluster headache” retrospective
“cluster headache” case series
“cluster headache” comparative study
Criteria for inclusion
1. Retrospective survey study on CH treatment
2. Quantifies and compares the self-reported efficacy of two or more substances in the treatment of cluster headache
3. Published between 2000 and 2020

Using the search function, a total of 994 articles were found. The inclusion criteria used to decide on the eligibility of the articles are listed in Table 1. Only articles quantifying the reported effects of two or more substances were included in the review as surveys on only one substance would have no common reference points with the other surveys. A time limit was considered necessary due to the development of treatment and survey methods, and the review was limited to articles published after the beginning of the year 2000. The selected time interval was chosen because the first online survey studying the perceived effects of CH medications was carried out in 2000 [29].

**Fig. 1:**
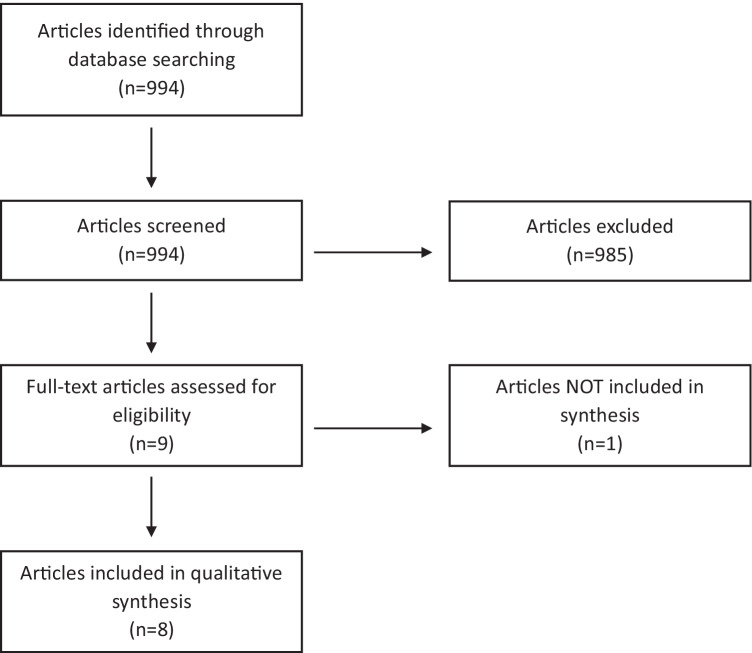
PRISMA flow-diagram outlining the study selection process

With every article, PubMed offers a list of similar articles and lists of articles commenting or citing the article in question, which were also utilized to complement the search. Also, the reference lists of the found survey articles were reviewed to find similar studies. However, no additional studies were identified through the complementary search methods. Nine articles were found to be eligible for full text assessment and selected for the systematic review based on all the selection criteria. The process of selection of studies is expressed through the PRISMA flow-diagram (Fig. 1).

### Data Collection

Following the final selection of the survey studies, two of the authors (SSR and SD) independently read through each of the selected surveys several times to identify factors which can be considered as possible biases in the surveys and to find variables for the data synthesis. Any disagreement was resolved with discussions. The factors identified included characteristics of the survey design such as the used survey instrument, how the survey was advertised (i.e., the recruitment channels used), the methods of verifying the CH diagnoses of the recruited participants, the aspects of the treatment focused on (e.g., the effectivity of only conventional treatments or also of unconventional ones, only effectivity or effectivity and availability), how the different treatments were categorized, and what parameters were used to describe different levels of treatment efficacy. In addition, the demographic and other characteristics of the participants (respondents) in the survey studies including age, location, and the ratio of chronic/episodic CH were extracted for each study. Finally, the self-reported abortive and prophylactic efficacy of established and unconventional treatments across different surveys was extracted for the data synthesis. The data collection mostly involved the articles and the respective supplemental files, except for the study by Schindler et al. [33] for which additional numerical data (which was only presented graphically in the article) were kindly provided by the authors.

### Data Synthesis and Analysis

The data concerning self-reported efficacies for acute or abortive treatments [29–37] were first combined in one table, and data concerning preventive or prophylactic treatments were combined into another table.

The scales used to measure efficacy were not the same in all articles. At first it was determined which of the studies had outcome variables that were ontologically similar enough to be used as such for the data analysis. Some surveys [29, 31, 35] had used a binary scale from the beginning such as effective/non-effective and responder/non-responder, whereas some [33, 36, 37] had reduced their original four or five level scale to a binary scale (i.e., two levels of efficacy) for analysis and presentation of their results. For the data analysis, outcomes from the first group of studies [29, 31, 35] were used as they were. For the second group of studies, the binary scales reduced from the four and five level categories by the original authors were used [33, 36, 37]. One study [30] had a three-part scale with an intermediate category of “partially effective,” between the effective and ineffective options, which was not possible to reduce into a binary scale and was used as such.

The synthetized combined data set of the self-reported efficacy of the treatments was explored with a standard data-driven hierarchical clustering. For the data from all of the 8 surveys, the efficacy of the treatments was considered as either “effective” or “not effective” and presented as binary data; however, in case of one survey [30], the three-level scale was presented as constricted binary data (i.e., partially effective responses were not included in either of the effective or non-effective response groups). The hierarchical clustering with Spearman’s rank correlation with average linkage was visualized as heatmap and dendrogram, which was created by using the Heatmapper-tool (http://www.heatmapper.ca/) implemented over R [38]. The hierarchical clustering arranged the self-reported efficacy of treatments into a tree, based on the correlation of the percentage of answers coded as “effective” and “not effective” across the different surveys.

## Results

### Characteristics of the Survey Studies and the Respondents

The characteristics of the survey studies and their respondents are summarized in Tables 2 and 3, respectively. Most of the studies mentioned providing the survey questionnaire to the respondents online [29, 32–36]. Although not always specifically mentioned, most of them used a structured questionnaire with closed-ended or multiple-choice questions providing specific response options to choose from. In one of the surveys [33], free text options were also provided for a few questions. Data collection method was mostly web-based (7 out of 9 surveys). Some studies targeted only CH support groups [29, 30, 32, 36], while the others targeted a wider population [31, 33–35, 37]. All surveys utilized convenience sampling. All studies aimed to ascertain the CH diagnosis of the respondents in the inclusion criteria, either through an additional interview, or having the diagnostic criteria included in the questionnaire, or by limiting the recruitment to neurology clinics. Four studies identified the respondents and verified the CH diagnosis; three studies did neither [30–35, 37].

**Table 2:** Characteristics of the survey studies

Study	N	Survey instrument	Recruitment channels	Ascertainment of CH status
Klapper et al. [29]	789	Internet-based survey with designed questionnaire. Mostly closed-ended questions on diagnostic criteria, family history of CH and treatments used	Non-clinic based (sufferers in general population) recruitment	Questionnaire included questions to confirm diagnosis based on IHS criteria
Sewell et al. [30]	53	Standardized questionnaire administered through interview	Recruitment through CH support groups and internet-based survey	Met ICHD-II criteria, then confirmed from their medical records documenting CH diagnosis by an expert
Schürks et al. [31]	246	Standardized questionnaires on demographics, CH characteristics, associated symptoms, CH diagnosis, lifestyle, details of treatment used	Clinic based (headache clinic) and non-clinic based (self-help groups or via the Internet) recruitment	CH diagnosis verified based on IHS criteria by direct history taking, or phone interview by neurologist, or mailed questionnaire
Di Lorenzo et al. [32]	54	Questionnaire developed by authors, administered through online interview or posted online. Questions on demographics, previous experience with conventional CH therapies, recreational use of illicit substances, and therapeutic use of illicit substances for CH	Recruitment through the Internet-based self-help group of CH patients	Diagnosed with CH by a neurologist, not validated by authors
Schindler et al. [33]	496	Online questionnaire based on existing literature. Mostly closed-ended; few with free-text options. Questions on demographics, CH characteristics, lifestyle, and treatments	Recruitment through CH websites, relevant online forums, and headache clinics	Responders reported being diagnosed with CH, verified by a neurologist or headache specialist
de Coo et al. [34]	643	Web-based questionnaire (or on paper) designed by the authors. Questions on lifetime use of illicit drugs, effects of illicit drugs on CH attacks	CH sufferers in general population and clinic-based recruitment	Diagnosed by validated web-based screening questionnaire about CH based on ICHD-II criteria
Rozen et al. [35]	1134	Online questionnaire developed by authors based on literature, cross-validated in test population. Multiple choice questions on CH clinical characteristics, triggers, treatment usage and efficacy, economics, family history, associated medical conditions, suicidality, headache related disability, tobacco, and alcohol use	Headache clinics, neurologist groups and organizations, CH support groups, and CH support group web site	Previously diagnosed with CH by a neurologist. The diagnosis of CH was not validated by the authors
Pearson et al. [36]	1604	Internet-based, closed-ended questionnaire designed by the authors. Questions on CH diagnostic criteria, effectiveness of treatments used, adverse effects, and accessibility of treatments	Recruitment through CH websites, relevant online forums, and headache clinics	Responders reported being diagnosed with CH by a medical professional. Also, questionnaire included questions to confirm diagnosis
Petersen et al. [37]	400	Survey questionnaire on CH developed by authors. Questions on treatments used, their effects on CH. Responses confirmed by semi-structured interviews	Recruited through headache clinics and online advertisements in relevant websites and newsletters	Diagnosed with ECH or CCH according to ICHD-II criteria, diagnoses verified by a headache specialist

**Table 3:** Characteristics of the responders in the survey studies

Study	CCH/ECH	M/F	Age (years)	Country of survey/targeted population	Smokers (%)	Respondent identified
Klapper et al. [29]	0.18	3.17	-	Survey published on the website of an US organization for general population	History of smoking: 77%	No
Sewell et al. [30]	0.66	3.82	AVG: 45.3	USA, the UK, the Netherlands and South Africa	-	Yes
Schürks et al. [31]	0.22	3.46	AVG: 44.8 (SD 11.5)	Europe (93% from Germany)	Current: 65.9 Former: 14.2	Yes
Di Lorenzo et al. [32]	1.16	1.84	-	Questionnaire published on the web page of an Italian self-help group	-	No
Schindler et al. [33]	0.47	2.82	MED: 41–50 *	USA	-	No
de Coo et al. [34]	0.31	2.72	MED: 49.9	Netherlands	53.8	Yes
Rozen [35]	0.31	2.57	MED: 41–50 *	USA	History of smoking: 73	No
Pearson et al. [36]	0.28	2.22	AVG: 46 (SD 13)	Survey was accessible online internationally. Major responders from the USA, the UK, and Canada	-	No
Petersen et al. [37]	0.59	2.03	AVG: 46.2 (SD 11.5), MED 47	Denmark	Current: 48.3 Former: 74.5	Yes

*CCH/ECH*, ratio of CCH patients to ECH patients; *M/F ratio*, ratio of male patients to female patients; *AVG*, average; *SD*, standard deviation; *MED*, median; (-) article provides no information; (*) only age groups reported

In total, 5419 respondents were included in the studies (Table 2). All studies included both ECH and CCH patients (Table 3). Seven of the studies had reported the average and median ages of the study populations which were between 40–50 years. Male to female ratio varied between 2.03 and 3.82, with older studies found to have higher male participants. Based on the reported ethnicity of the respondents, the majority was of European descent from Europe or North America (Table 3). Five surveys probing on smoking habits of the respondents reported majority of them (53.8–77%) as current or former smokers. Regarding the ratio of CCH patients to ECH patients, five surveys had reported roughly similar ratios (between 0.18 and 0.31) [29, 31, 34, 35, 37]. However, in the rest of the surveys, the ratio ranged between 0.47 and 1.16 with the highest being in the study by Di Lorenzo et al. [30, 32, 33, 36].

### Assessment of CH Treatments in the Survey Studies

**Fig. 2:**
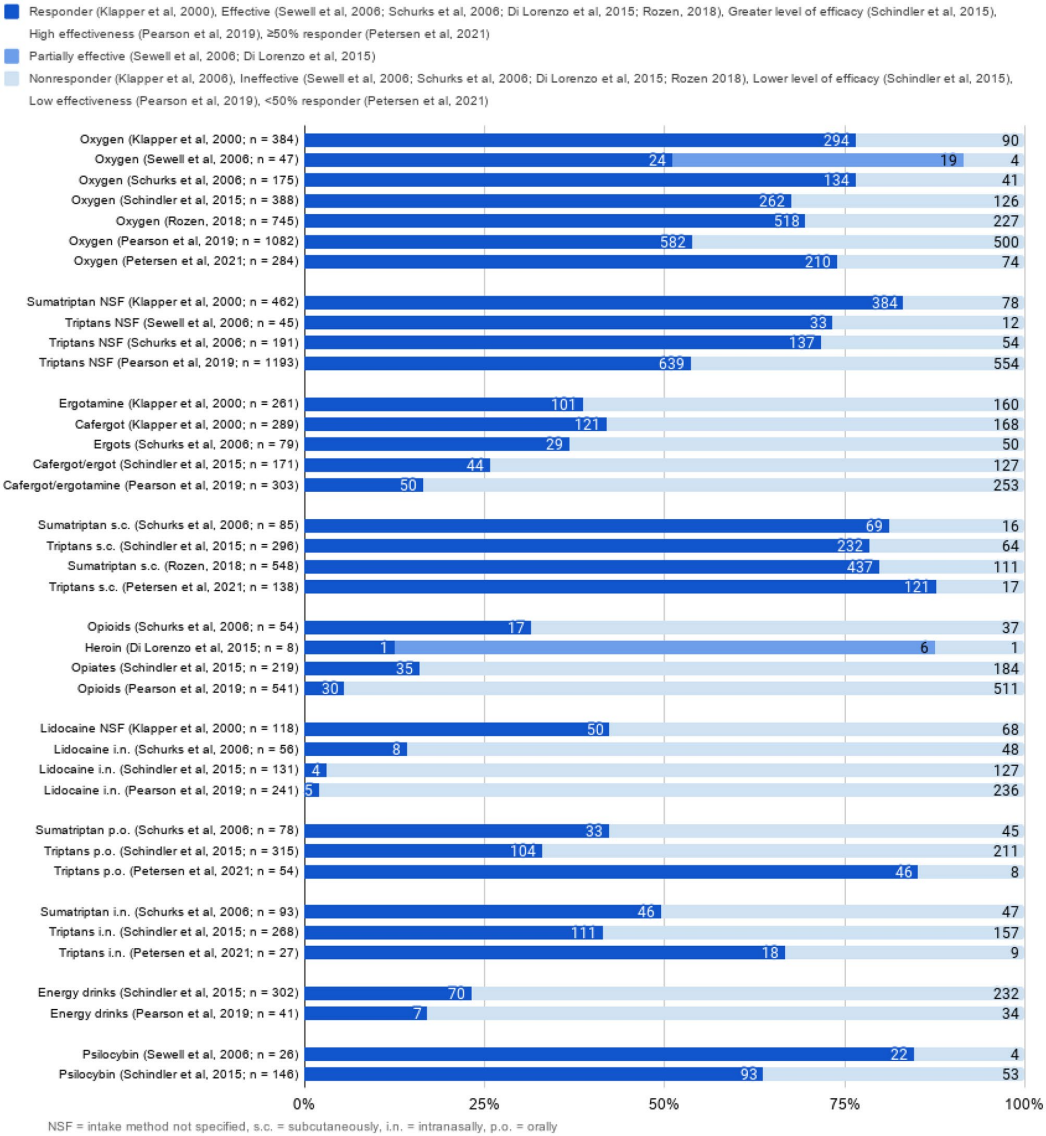
The combined data for the reported effectiveness of different treatments for acute CH attack abortion. The figure includes results from all 8 included surveys. The data is represented in binary scale (effective, not effective) when possible, or in a scale including partial effectiveness in case of two articles

**Fig. 3:**
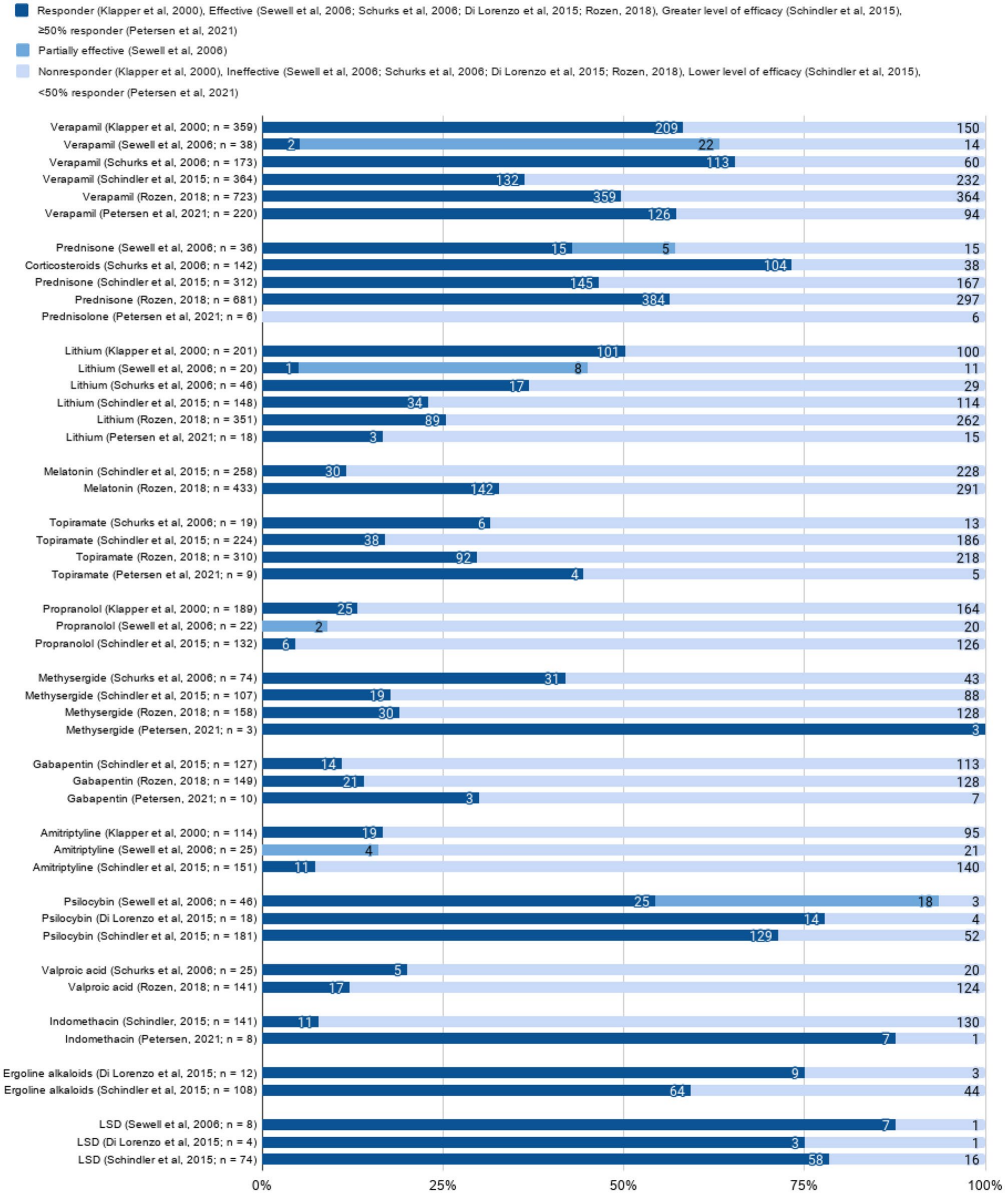
The combined data for the reported effectiveness of different treatments for acute CH attack abortion. The figure includes results from all 8 included surveys. The data is represented in binary scale (effective, not effective) when possible, or in a scale including partial effectiveness in case of two articles

Apart from inquiring about the efficacy of the treatments, treatment types, reasons for selecting specific treatment, treatment accessibility, and association of treatment use with specific clinical features were also probed (Table 4). None of the surveys included questions that reported direct preference between any two treatments. Of the nine surveys, two specifically focused on the use of unconventional treatments [32, 34], and two asked about both conventional and unconventional treatments [30, 33]. The rest of the surveys mainly focused on conventional treatments, with one focusing mainly on oxygen [36] (Table 4). Eight out of the nine included surveys inquired whether the respondent would label a treatment as highly effective or not effective, or something in between using two (i.e., binary) to five level scales. Thus, in these eight studies, the self-reported efficacy was measured in a way that made it possible to present the results in parallel (Figs. 2 and 3). [298, 30, 31, 32, 33, 35, 36, 37]. The study by de Coo et al. [34] specifically inquired about the effects on both the duration and frequency of the symptoms, while the other studies inquired only about the general effects on the symptoms. Because of this marked difference, the results from this study were excluded from the data synthesis step (presented in Figs. 2 and 3).

**Table 4:** Assessment of CH treatments in the survey studies

Study	Treatments asked about	Treatments categorized as	Parameters to assess treatment effectiveness	Definition of effectiveness
Klapper et al. [29]	Treatments used: • Effectiveness • Accessibility	Known abortive and preventive medicines Medicines not proven to be effective Other: Surgery and radiology	Responder, non-responder	-
Sewell et al. [30]	Illicit drug used: • Type (psilocybin-containing mushrooms or LSD) • Mode • Effectiveness Conventional drug use	Acute Prophylactic Remission extension	Effective, partially effective, ineffective	Effective in aborting attacks: ending attacks in 20 min Effective prophylactically during a cluster period: causing total cessation of attacks Partial efficacy in prophylaxis: attacks decreasing in intensity or frequency but not ceasing Extension of remission: next expected cluster period delayed or prevented entirely
Schürks et al. [31]	Treatments used: • Choice • Effectiveness	Acute and prophylactic medication Use of first-line medication for treatment of CH attacks and prophylaxis Use of experimental and traditional pain medications	Effective, not effective	Effective if effectiveness had been established ≥ 3 times Effective acute treatment: if it reduced pain by at least 50%, at least 50% of time within 15 min of sub-cutaneous application or 30 min of other application forms, compared to untreated attacks Preventive medication effective if CH episode terminated or attack frequency reduced by at least 50% within 2 weeks
Di Lorenzo et al. [32]	Illicit drug used: • Types • Reason for use • Effectiveness Conventional drug use: • Experience • Effectiveness	Abortive Prophylactic	Effective (fully or in part), Ineffective, Worsening	-
Schindler et al. [33]	Conventional and illicit drug used: • Effectiveness	Abortive and preventive medications Effects on cluster period, remission, and conversion	Completely effective, moderately effective, partially effective, not effective	-
de Coo et al. [34]	Illicit drug used: • Effectiveness on CH attack duration and frequency	CH attack treatment	CH attack frequency and duration: Decrease, no effect, increase, unknown	-
Rozen [35]	Treatments used: • Effectiveness	Acute Preventive	Effective Non-effective	-
Pearson et al. [36]	Oxygen used: • Effectiveness in comparison to other acute treatments	Preventive medications Abortive medications Unregulated treatments Surgical/neuromodulation treatments	Completely ineffective, minimally effective, somewhat effective, very effective, completely effective	-
Petersen et al. [37]	Treatments used: • Types • Association with specific clinical features • Effectiveness	Current and previous acute medications	Effect rated for current treatment only: “Completely, the pain is gone,” “Some, the pain is halved or more,” “A little, it only takes the top off the pain,” “None, the pain is unchanged”	For acute treatment: 50% responders were patients who reported “completely, the pain is gone” or “some, the pain is halved or more” 100% responders were patients who reported “completely, the pain is gone” For preventive treatment: same as for acute

**Fig. 4:**
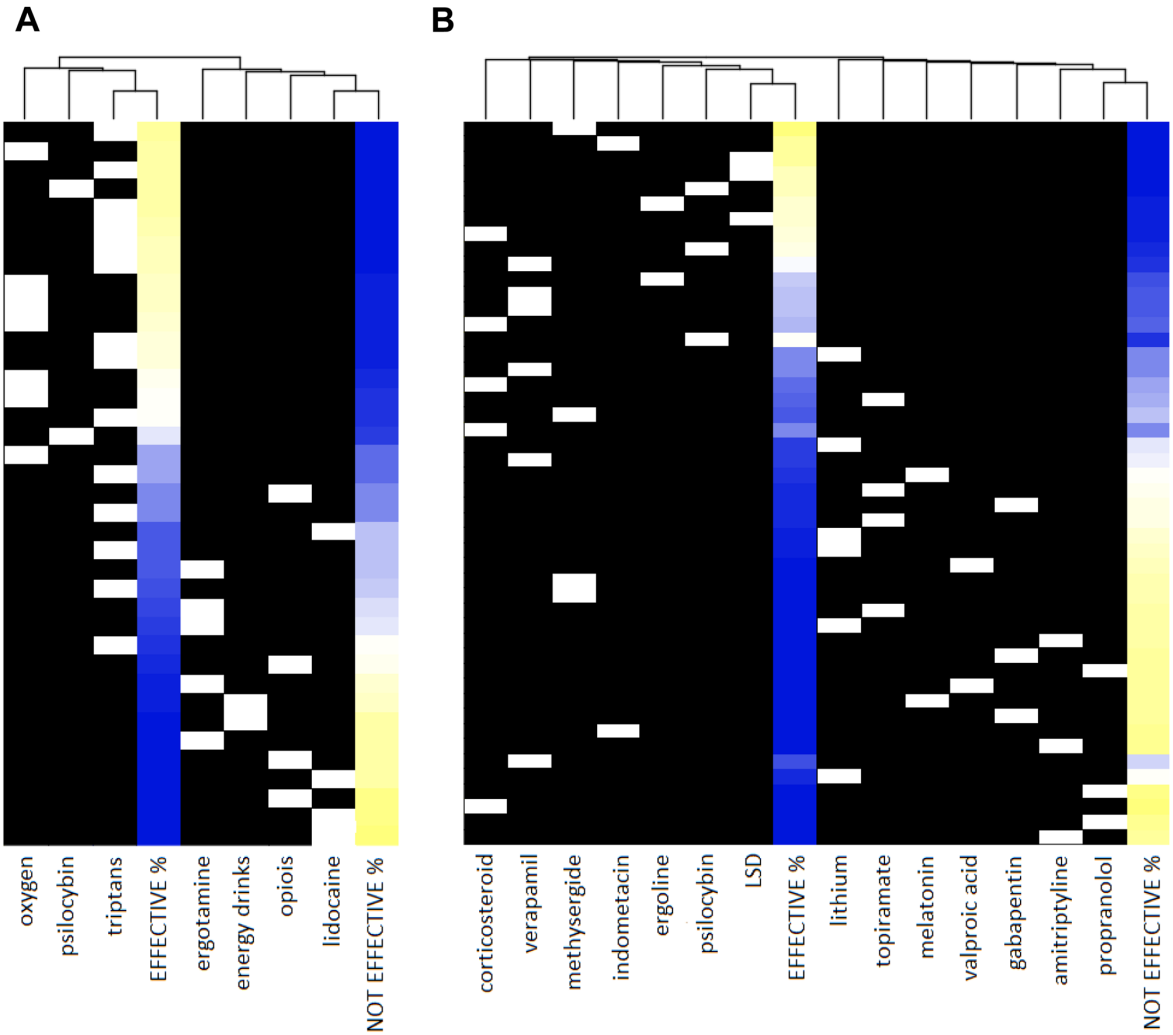
The hierarchical clustering of self-reported efficacy of the treatments, Spearman correlation with average linkage. Each line consists of: white marker, each indicating the place of a single treatment from a single survey. There are multiple white markers per column, one for each survey which reported on the same treatment. The blue-to-yellow gradient indicates the frequency of both the self-reported effective and self-reported not-effective responses for one treatment from one survey per line. Dark blue indicates frequency of 0%, and bright yellow indicates frequency of 100% of the participants. The columns are organized based on the hierarchical clustering algorithm and both the treatment and frequency columns are placed based on the rank correlation between the columns, as indicated by the dendrogram on the top of the figure. The exact place of the column has no meaning, the place in the dendrogram is the sole indicator for the similarity of any columns as determined by the correlation between the treatments and the frequency data

The scales used in the eight included studies varied in the number of options and words expressing the levels of the scales. Three studies [29, 31, 35] used binary scales to collect and analyze their data on treatment efficacy. Three others [33, 36, 37] used a four or five level scale in data collection but reduced to binary scales for data analysis. In our study, we have analyzed and presented these self-reported treatment efficacies in a binary scale of “effective” and “not effective” options (Figs. 2, 3 and 4). The “effective” option included “responder” [29], “effective” [31, 35], “greater level of efficacy” [33], “high effectiveness” [36], and “ ≥ 50% responder [37]. The “not effective” option included “non-responder” [29] and “ineffective” [31, 35], “lower level of efficacy” [33], “low effectiveness” [36], and “ < 50% responder” [37]. One of the studies [32] had a three-level scale of “effective,” “non-effective,” and “worsening” options. In our analysis, we included “worsening” also in the “not effective” option. Another study [30] had a three-level scale of “effective,” “partially effective,” and “ineffective” options. The “effective” and “ineffective” were included in the options of “effective” and “not effective,” respectively. However, the “partially effective” option closely resembled the description of the “effective” category of the articles that described their scales, but as only some of the articles described their scales, it was not considered feasible to report the results of this study [30] in binary form (Figs. 2 and 3) and hence was presented as such. The “partially effective” option was, however, omitted from the hierarchical clustering (Fig. 4) to avoid artifacts.

### Self-Reported Efficacy of the Surveyed CH Treatments

There are marked differences in the reported efficacies of different treatment substances (Figs. 2 and 3), and the reported efficacy of the treatments is generally consistent across the different studies (Fig. 4). A rank-order hierarchical clustering was created to inspect the consistency of the self-reported efficacies between the survey studies. The abortive treatments fell into two clades based on their efficacy. Oxygen, triptans, and psilocybin belonged to the same clade. Both oxygen and psilocybin were reported as effective by the respondents in multiple surveys. For triptans, there was less consistency due to the lesser self-reported efficacy of oral triptans (Figs. 4A, 2).

For the prophylactic effect, LSD, psilocybin mushrooms, ergoline alkaloids, methysergide, verapamil, and corticosteroids belonged to the clade with higher correlation to self-reported efficacy in the rank-correlated dendrogram with average linking (Fig. 4B). Melatonin, valproic acid, and gabapentin, and especially amitriptyline and propranolol, belong to the clade of treatments with low self-reported efficacy, with consistent low efficacy (Figs. 4B, 3). The consistency for the self-reported efficacy can be seen only for LSD, psilocybin mushrooms, and ergoline alkaloids, and the highest frequency for prophylactic efficacies was also reported with the use of these serotonergic substances [30, 32–34] (Fig. 3). Corticosteroids were reported to be the most efficient conventional prophylactic treatment, followed closely by verapamil (Fig. 3). The most marked deviations from the mean were in the results of Petersen et al. [36], in treatment groups where the number of users was less than ten (Figs. 2 and 3).

All the aforementioned directly acting serotonergic substances were reported to be more effective than verapamil and even corticosteroids, also in studies where these were compared [30, 33]. Psilocybin mushrooms were also reported to have an abortive efficacy comparable to oxygen and subcutaneous triptan injections [23]. In comparison to verapamil, lithium, corticosteroids, and other conventional medications, 5-HT2A agonists such as psilocybin mushrooms and LSD were often reported to induce a long-lasting reduction or a complete cessation of symptoms with only a single dose [30, 32, 33]. While these substances are acutely psychoactive at certain doses [39, 40], a substantial preventive treatment response was also reported with small, non-psychoactive doses [30, 33]. Side effects were reported to be uncommon and minor [33, 34].

In the case of Sewell et al. [30], omitting the “partially effective” responses due to the lack of common reference points produced two outliers in the preventive group, with verapamil and lithium seemingly underperforming in that survey study compared to the rest. Based on the descriptions of the scales in the original studies, the “partially effective” category of Sewell et al. [30] closely resembles the “effective” category of Schürks et al. [31] and Petersen et al. [37]. As seen in Figs. 2 and 3, assigning “partially effective” responses from Sewell et al. to the “effective” option would have matched almost exactly with the results from other surveys, also in case of oxygen and psilocybin. However, the constricted binary scale used in the dendrogram is justifiable as the order of the effectiveness of the treatments (Fig. 4) were consistent in that survey [30] as compared to other surveys.

Differences between the treatment efficacies in ECH and CCH patients were evaluated in four of the surveys. Statistically significant differences were reported only for oxygen and triptans, which were both considered more effective in the ECH group [31, 35–37].

## Discussion

The most important findings of this review were the consistency of the survey results regarding the self-reported efficacy of CH treatments and the marked differences between the reported efficacies of different substances. In addition, psilocybin mushrooms and LSD, both centrally acting 5HT-2A-agonists, were most reported as effective prophylactic treatments in CH. The findings of this review also showed that if the limitations of the survey studies are taken into consideration, combining data from multiple survey studies can provide important and reliable information. Thus, results of this review are not only useful to understand the current state of CH treatment from the perspectives of the patients, but also in providing information to aid future CH research.

### Consistency Between the Survey Results and Close Agreement with the Clinical Data

There was overall good consistency in the reported efficacies across the 8 surveys, despite methodological differences. There were consistent and marked differences between the reported efficacies of different treatments across 8 survey studies, and the order of efficacy is generally in agreement with clinical data [1, 2, 4–7]. Based on clinical practice and data produced by clinical studies, oxygen, triptans, verapamil, and corticosteroids are widely considered the most effective CH treatments [1, 2, 4–7]. These were reported to be the most effective conventional treatments also in the surveys reviewed here. In our study, injectable sumatriptan was found to be consistently reported more effective than orally or nasally administered triptans, which is also in accordance with the findings of clinical studies [4, 7]. Low level of efficacy was reported for valproic acid, gabapentin, indomethacin, amitriptyline, and propranolol, none of which are suggested for CH treatment in recent recommendations [1, 2, 4–7].

### High Self-Reported Efficacy of Psilocybin and LSD in the Prophylactic Treatment of CH

An interesting theme detected in our results is the consistently self-reported high efficacy of psilocybin mushrooms and LSD as preventive treatments [30, 32–34]. These results are also echoed in the excluded survey study by de Coo et al. [34] and other articles on the subject, such as a case report by Sempere et al. [41], a small case series by Karst et al. [9], and a thematic analysis of online discussions by Andersson et al. [42]. An exploratory controlled study by Schindler et al. [55•] found that the single administration of a low dose of psilocybin produced a significant and long-lasting reduction in migraine symptoms. Placebo response in CH is considered relatively small [44•], and it is unlikely that it alone would explain the results. It should however be noted that the combined number of respondents reporting the use of these substances in our study was small in comparison to the number of patients using conventional treatments.

The primary common factor between LSD and psilocybin is their activity in central serotonin 2A (5HT-2A) receptors [39, 40]. Structurally, LSD resembles the ergolines used in the treatment of CH, whereas psilocybin resembles triptans [39, 40, 43]. Psilocybin is most commonly consumed in the form of psilocybin mushrooms; the primary serotonergic alkaloids present being psilocybin and psilocin. Other tryptamines in the mushroom include norpsilocin, baeocystin, norbaeocystin, and 4-hydroxytryptamine, which are present in smaller amounts [56] but may also have some biological activity. Possible therapeutic mechanisms of psilocybin and LSD have been reviewed by Schindler et al. [44•].

### Biases in the Survey Studies

Survey studies are prone to several biases [35, 36]. The survey technique, the population studied, the responses, and the analysis might all be biased. Each of the included articles described their respective limitations. The assessment of the risk of bias of the individual surveys in our study is based primarily on the extracted variables presented in the results section (Tables 2, 3 and 4) which represent several central characteristics of a CH patient population [1] and key characteristics necessary for assessing biases and shortcomings commonly present in questionnaire studies [35, 36]. All reviewed surveys were retrospective, and most were online questionnaires open for anyone to respond to. Patients were identified only in four of the nine surveys [29, 31, 34, 37]. The diagnosis was confirmed from medical records or by a specialist only in the surveys made by Sewell et al. [30] and Petersen et al. [37], making the rest of the surveys depended upon the veracity of the respondents. Every study except the survey by Petersen et al. [27] listed patient support groups as a source of recruiting respondents. Hence, it is possible that some participants have responded to two or more of the surveys. It is possible that patients involved in support groups are less satisfied with their treatment than patients outside these groups, making the conventional treatments appear less effective than in the general CH population. Some patient groups also openly endorse certain alternative treatment methods, such as the use of psychoactive tryptamines or ergolines (also known as “busting”) [30, 32–34].

It did not seem that the survey respondents were entirely representative of the wider CH population in any of the surveys. The male to female ratio (2.03–3.82) and reported average and median ages (between 40 and 50 years) were somewhat similar to the general CH population (male to female ratio is about 3, average age of onset about 30). However, the ratio of CCH to ECH patients was higher in the survey respondents when compared to the general CH population [1], which may indicate that due to the difficulty in treating CCH patients, they are more interested in contributing in the CH related research. It is likely that few or none of the respondents had used all the treatments listed in the survey, nor there were questions about direct comparison between two or more treatments.

A major source of bias in this review was the different scales of efficacy used in the surveys. The observed consistency of the results is strongest in the studies using similar binary scales [29, 31, 35]. The reduced binary scale of Pearson et al. [36] is uneven and probably makes the “low effectiveness” category seem artificially larger than the similarly reduced scales of Schindler et al. [33] and Petersen et al. [37]. This is supported by the observation that the efficacy of any specific comparable substance is reported to be greater in the results of Schindler et al. [33] and Petersen et al. [37]. The three-level scale by Sewell et al. [30] was not reduced to a binary scale in data synthesis. In that study, however, the “effective” category is very strict, and the “partially effective” category [30] is similar to the “responder” and “effective” categories in binary scales of Klapper et al. and Schürks et al. [29, 31].

### Strengths and Limitations of Combining Survey Data

To increase the value of the surveys, the survey results should be analyzed together, but no standard method for combining their results exists. There are benefits of analyzing together data collected from multiple locations, populations, and produced with several methods [45, 46]. Multiple data sources for the same phenomenon would increase the robustness of interpretation [47–49]; the diversity of survey designs and different settings do not weaken but increase the confidence that the observed phenomenon is not an artifact of a single study setting [46, 48, 50–52].

Application of multiple “reasoning strategies” [45, 46] can help to elaborate the strengths and weaknesses of our results. Foremost, considering the strength and the consistency of the effect [50], the clinically most effective conventional abortive treatment, oxygen, was also found to be most reported as efficacious in all the reviewed studies. The results match the overall pattern [52] of the expected rank order of the treatments based on literature. In this review, LSD and psilocybin mushrooms, widely used in self-treatment [30, 32–34], were found in the same dendrogram clade as the most efficacious conventional treatments. Also, their self-reported prophylactic efficacy reached ~ 75% frequency, highest among all the prophylactic treatments surveyed, indicating an association between the self-administration of these substances and CH attack prevention.

There were no results disagreeing with the current knowledge. The present results are also plausible, as psilocybin mushrooms, LSD, ergolines, and triptans, all have similar serotonergic mechanisms of action. Psilocybin and LSD penetrate the blood–brain barrier with ease, increasing their potential for central activity as compared to triptans and other serotonergic medication, which might help to better understand the reported long-lasting effects of single doses [43]. 5HT-2A-receptors are prominently expressed in hypothalamus, which is considered important for the pathogenesis of CH [3, 43, 53]. The results of this review can, therefore, be used to form a hypothesis that 5-HT2A agonists such as psilocybin and LSD, due to their central action, can be efficacious in CH attack prevention. The results strongly suggest that the reasonably well-tolerated [39, 40] psilocybin is a promising lead to be systematically clinically tested as a prophylactic CH treatment. The first RCT of psilocybin in CH has completed, and the report is pending (NCT02981173).

There are aspects that surveys do not provide answers to. Biological gradients, for example, dose-responses, were not established in the surveys. The specificity of outcome variable could not be ascertained in this review as surveys did not report data on the combination of treatments. The surveys also did not report direct comparisons between the self-reported efficacy of two or more treatments. None of the surveys considered how many treatments the patients were using previously or at the time of the survey.

There is a valid analogy of an unconventional self-treatment becoming accepted and even recommended as a first-line treatment for CH. Oxygen was serendipitously discovered and has been used as a self-treatment for CH since the 1930s, but RCTs were not conducted until the 1980s [5, 54]. Oxygen is now considered to be one of the most effective abortive CH treatments [4, 7]. Most of the substances used to treat CH were originally developed for another purpose, and trying unconventional substances for the treatment of CH is not a new suggestion [5].

### Future Considerations

Future survey studies should utilize random sampling to form samples that are more representative of the general CH population and also target different ethnic groups. Identifying the respondents and verifying their diagnosis would give the results more credibility. Adverse effects should be measured in addition to therapeutic effects. Questions which ask the participant to directly compare the self-reported efficacy between any two treatments should be utilized, as these questions allow the combined estimations of the efficacy of multiple treatments across multiple surveys similarly to classic meta-analyses. Also, prospective surveys and cohort studies should be performed. There is no simple answer to what the precise measurement scales should be like, but the scales should at least be defined clearly.

### Relevance in Clinical Practice

This review highlighted that complementary or alternative treatments for CH are endorsed by many patient groups and are commonly used by CH patients in self-treatment [13–18, 21, 30, 32–34]. This finding indicates that the patients may fail to receive adequate support from their physicians, which may have forced them to take the path of self-treatment or use of unconventional treatments without any professional supervision. Hence, clinical practitioners would require the knowledge on these alternative treatments to openly discuss the possible harms and benefits and evaluate contraindications and interactions with the patients who use unconventional treatments [13, 14, 32]. The preliminary positive results on some substances might help physicians understand why some patients would want to use them. On the other hand, patients’ perceptions on the subject might be incorrect or biased. The current evidence on CH treatments, as presented in this review, therefore, would aid the physicians to communicate and educate their patients.

## Conclusions

In this first systematic review of retrospective survey studies on CH treatment efficacy, we gained critical information on the current state of CH treatments from the perspectives of CH patients. Survey studies are valuable to assess the experiences of the CH patients and to guide and complement clinical studies. We observed consistency in the reported efficacy of most treatments across the surveys and consistent differences between the reported efficacies of different substances used for CH treatment. The reported order of efficacy of the studied treatments was in accordance with clinical studies.

Although surveys are known to have methodological shortcomings and biases, multiple reasoning strategies indicate that a new robust phenomenon; i.e., the frequent and consistent high self-reported efficacy of psilocybin mushroom and LSD in prophylactic treatment of CH was captured in this review. This phenomenon seems to explain why an estimated one-third of the CH patients use “unconventional” treatments, despite the social taboo and legal sanctions attached with the use of these substances. The mechanism of action of these serotonergic substances also supports their efficacy in the treatment of CH. The findings of this review, therefore, strengthen the case for assessing the efficacy of 5-HT2A agonists in prospective RCTs.

## Supplementary Information

Below is the link to the electronic supplementary material.

Supplementary file1 (DOCX 170 KB)

## Data Availability

All data analyzed during this study are from articles used as references 28 to 36.
