# Supplementary material for: Self-Reported Efficacy of Treatments in Cluster Headache: a Systematic Review of Survey Studies
Source: Curr Pain Headache Rep. 2022 Jun 27;26(8):623–37. doi: 10.1007/s11916-022-01063-5 (PMC9436841; doi:10.1007/s11916-022-01063-5)
Supplement: Supplementary file 1 — Supplementary file1 (DOCX 170 KB) [file 11916_2022_1063_MOESM1_ESM.docx]

The produced search function was:

“("cluster headache"[All Fields] AND ("survey s"[All Fields] OR "surveyed"[All Fields] OR "surveying"[All Fields] OR "surveys and questionnaires"[MeSH Terms] OR ("surveys"[All Fields] AND "questionnaires"[All Fields]) OR "surveys and questionnaires"[All Fields] OR "survey"[All Fields] OR "surveys"[All Fields])) OR ("cluster headache"[All Fields] AND ("questionnair"[All Fields] OR "questionnaire s"[All Fields] OR "surveys and questionnaires"[MeSH Terms] OR ("surveys"[All Fields] AND "questionnaires"[All Fields]) OR "surveys and questionnaires"[All Fields] OR "questionnaire"[All Fields] OR "questionnaires"[All Fields])) OR ("cluster headache"[All Fields] AND ("censused"[All Fields] OR "censuses"[MeSH Terms] OR "censuses"[All Fields] OR "census"[All Fields] OR "censusing"[All Fields])) OR ("cluster headache"[All Fields] AND "poll"[All Fields]) OR ("cluster headache"[All Fields] AND ("inquiries"[All Fields] OR "inquiry s"[All Fields] OR "research"[MeSH Terms] OR "research"[All Fields] OR "inquiry"[All Fields])) OR ("cluster headache"[All Fields] AND ("interview"[Publication Type] OR "interviews as topic"[MeSH Terms] OR "interview"[All Fields])) OR ("cluster headache"[All Fields] AND ("comparison"[All Fields] OR "comparisons"[All Fields])) OR ("cluster headache"[All Fields] AND ("retrospective studies"[MeSH Terms] OR ("retrospective"[All Fields] AND "studies"[All Fields]) OR "retrospective studies"[All Fields] OR "retrospective"[All Fields] OR "retrospectively"[All Fields] OR "retrospectives"[All Fields])) OR ("cluster headache"[All Fields] AND "case series"[All Fields]) OR ("cluster headache"[All Fields] AND "comparative study"[All Fields])”.
